# Techno-emotional projection in human–GenAI relationships: a psychological and ethical conceptual perspective

**DOI:** 10.3389/fpsyg.2025.1662206

**Published:** 2025-09-29

**Authors:** Chiara Saracini, María Isabel Cornejo-Plaza, Roberto Cippitani

**Affiliations:** ^1^The Neuropsychology and Cognitive Neuroscience Research Center (CINPSI Neurocog), Faculty of Health Sciences, Universidad Católica del Maule, Talca, Chile; ^2^Neurometa and IA+D, Research Group, Universidad Autónoma de Chile, Santiago, Chile; ^3^Innovation, Technology and Frontiers of Legal Science Laboratory (Lisa Lab), Universidad Autónoma de Chile, Santiago, Chile; ^4^Department of Law, Università degli Studi di Perugia, Perugia, Italy; ^5^Facultad de Derecho, Cátedra ISAAC (Individual Rights in Scientific Research and Cooperation), Universidad Nacional de Educación a Distancia, Madrid, Spain; ^6^INDEPAC—Instituto Nacional de Estudios Superiores en Derecho Penal, Mexico City, Mexico

**Keywords:** generative artificial intelligence, human-AI interaction, techno-emotional projection, psychological dynamics, AI ethics

## Abstract

Generative artificial intelligence (GenAI) is being increasingly integrated in everyday applications and devices. In this new frontier of technology interface, psychologists hold a significant role in understanding and guiding human-AI interactions. This theoretical contribution proposes a framework for the psychological and ethical dimensions of human–GenAI relationships, structured across four key dimensions: (1) individual psychological characteristics and susceptibilities, such as insecure attachment styles, low self-efficacy, and emotional dysregulation or immaturity; (2) interpersonal dynamics, including emotional projection and the illusion of reciprocity; (3) processes occurring at the group level, such as the symbolic inclusion of GenAI agents within human communities or social groups and the evolution of societal norms; and (4) emerging ethical concerns, such as perceived agency, illusory consent, and the use of synthetic data that may amplify biases, alongside the utilization and acquisition of biometric and cognitive data for interaction modeling. Within the sphere of interpersonal dynamics, we propose the concept of “Techno-Emotional Projection” (TEP) to describe how emotionally vulnerable users may project relational needs onto emotionally responsive but non-conscious technologies. This projection can lead to a sort of “emotional looping” (a recursive reinforcement of expectations through repeated interaction) and, over time, to the formation of a synthetic attachment to the GenAI technology. Drawing from psychological theories and empirical studies, we argue that these relationships have subjectively real consequences and deserve careful study. Finally, we propose directions for ethical design, emotional AI literacy, and socially responsible integration of GenAI into human life. This perspective aims to foster a balanced, informed, and human-centered approach to this rapidly evolving field.

## 1 Introduction

The emergence of Generative Artificial Intelligence (GenAI), including large language models (LLMs), emotionally responsive chatbots, and socially interactive robots, has reshaped the way we conceive human-technology interaction. Beyond their role as functional tools or information assistants, these systems are increasingly perceived as social actors capable of engaging in emotionally meaningful exchanges (Lee and Nass, 2010; Nass and Moon, 2000; Reeves and Nass, 1996). Through natural language, adaptive responses, embodied avatars, and personalized feedback, GenAI systems simulate behaviors typically associated with human intimacy, empathy, and emotional care (Brooks, 2021; Kirk et al., 2025; Turkle, 2011, 2024). This shift moves AI from being perceived as purely utilitarian (a technological tool) to being experienced as a potential social actor. The design of current AI-based systems not only facilitates practical interactions, but also encourages complex emotional and symbolic engagement (Følstad and Brandtzæg, 2017; Turkle, 2011). Over the past decade, general-purpose AI has advanced from narrow task automation to increasingly human-like conversational and adaptive systems. This rapid acceleration has initiated a transition, fostering the growing perception that AI agents are entities to relate with, rather than tools to be used. The legacy of the Turing Test reinforced the idea that linguistic indistinguishability from humans represents the benchmark of machine intelligence (Turing, 1950, 2009). While GenAI systems demonstrate sophisticated linguistic capabilities, reportedly able to pass the Turing’s Test (Jones and Bergen, 2025), philosophical and neuroscientific literature clearly distinguishes behavioral simulation from genuine consciousness or intentionality (Chalmers, 2016; Searle, 1980). The ability to process and generate human-like language does not necessarily indicate understanding, self-awareness, or subjective experience (Block, 1995; Dreyfus, 1992). This anthropomorphic bias (or “anthropomorphic fallacy”; Placani, 2024), rooted in the assumption that successful imitation implies deeper cognitive equivalence (in the “imitation game” that will eventually make the machine evolve into a cognitive-like system; Turing, 1950, 2009), leads many to interpret a machine that can “speak like a human” as capable of understanding and empathizing, thereby humanizing what is ultimately an algorithmic system. This tendency to interpret all forms of intelligence and interaction through a human-centered lens might be not only misleading, but also limiting. In attributing human-like qualities to GenAI, we may not be uncovering its true nature, but rather projecting the frameworks by which we understand ourselves. As we know from research on anthropomorphism and social cognition, this anthropomorphic projection (attributing intentionality, agency and emotion even where none exists) is amplified when entities display human-like cues such as language, faces, or emotional feedback (Epley et al., 2007; Waytz et al., 2010a; Waytz et al., 2010b). In this sense, our interpretation of GenAI may reveal more about human cognitive and affective biases than about the technology itself. A simple individual cognitive bias might become a sociocultural issue as the rapid proliferation of GenAI technologies translates these interpretative tendencies into concrete psychological, societal and ethical consequences.

The rapid development of new GenAI-based technologies, in this scenario, needs an urgent and deep psychological examination, as the transition from a tool to an interactive companion reshapes users’ expectations, attachment patterns, and sense of reciprocity in ways that were once exclusive to human–human relationships. Such relational dynamics are not psychologically neutral; they can shape cognition, emotion, and behavior, raising urgent psychological, ethical, legal and social questions. What kind of relationship is a person developing when they are emotionally connected to a non-conscious, non-biological “entity”? Can such relationships fulfil genuine psychological needs, or do they risk deepening emotional dependence and disconnection from human communities? As millions of users worldwide engage daily with GenAI systems such as ChatGPT, Replika, or therapeutic chatbots (e.g., Woebot, Wysa), we are moving beyond HCI-based utility models developed during the past century and many people are possibly experiencing these interactions as “interpersonal” and potentially ethically charged relationships.

### 1.1 The rapid evolution of AI capabilities toward human-like interaction

Over the past decade, there has been a remarkable transformation in artificial intelligence systems. What began as rule-based programs designed to efficiently complete narrow tasks has expanded far beyond the laboratory setting into a vast range of commercially available applications, including search engines, facial recognition systems, customer service chatbots, medical diagnostic tools, and autonomous vehicles, with new uses emerging at an accelerating pace (Jacobides et al., 2021).

The most striking shift has been the rise of generative AI, which no longer relies solely on rule-based logic but is capable of producing complex, contextually relevant outputs. Modern generative models can generate seemingly emotional responses and sustain personalized conversations over extended periods (Brown et al., 2020).

They are also increasingly able to recognize affective cues in text or voice, adapt communication styles to individual users, and engage in dialogues that feel remarkably similar to human-to-human interaction (Kapase and Uke, 2025). As a result, users often report forming emotional bonds with AI chatbots—turning to them for comfort in times of distress and overcoming loneliness, even describing these relationships in terms of friendship or love (Brooks, 2021; Følstad and Brandtzæg, 2017; Kirk et al., 2025). This evolution marks not merely a quantitative increase in functionality, but a qualitative transition in the very nature of human–technology interaction.

We can foresee at least 2 directions that human relationships with GenAI might take in the short future: (a) relationship with an interface, such as chatGPT o chatbots, which are basically LLMs, now being enriched with customizable voice or avatars (like in character.ai; Sharma et al., 2025) and (b) relationship with humanoid AI robots (language models with a body and emotional intelligence), such as “Eliza Wakes Up” (elizawakesup.ai) or “Aria”,^1^ explicitly made to overcome loneliness and interact in an intimate and personal way, designed as “companion robots,” or cooperative and educational robots such as Cozmo (Lefkeli et al., 2021). While the first type is already accessible to the general public via laptops and smartphones, the second remains expensive and less widespread, though likely to become more common in the future. In both cases, however, the psychological responses they evoke are similar: users project emotional needs and expectations onto the AI counterpart. They are thought to be able to *“fill the emotional void”* and *“tackle the staggering loneliness epidemic”* haunting our modern societies (Collins, 2025; Murthy, 2023). We will discuss mainly about the first class of GenAI relationships, because they are the most studied, but we anticipate that the concerns raised might be even doubled in the case of the second kind of artifacts.

These dynamics reflect broader societal trends in which technology plays an increasingly important role in mediating culture, relationships and emotional life (Erstad, 2025). From video games to social media, new forms of digital dependency have emerged to fulfil unmet emotional needs (Kuss and Griffiths, 2017; Ryan et al., 2014). In general, addictions and dependencies can originate from early developmental failures in affect and emotional regulation, where emotional deprivation and insecure attachment patterns increase vulnerability to compulsive behaviors and addictive cycles (Alvarez-Monjaras et al., 2019). The intense use of GenAI that we see today may, to some extent, represent just another technology-mediated addiction (a topic that will certainly also be at the center of mental health debates and research in the next decades). This might be the case when users exhibit addictive patterns and behaviors towards chatbots and crave their daily chatGPT conversations. However, GenAI relationships in certain cases can also potentially be disruptive in the presence of non-addictive behavior, as we will discuss later. Unlike problematic internet use or social media addiction, which involve human-to-human interaction mediated by technology, GenAI introduces direct emotional engagement with non-conscious agents. Even in the case of parasocial relationships, characterised by one-sided projections onto static media figures (Hartmann and Goldhoorn, 2011; Horton and Wohl, 1956), there is a crucial difference: GenAI provides adaptive, dynamic feedback, creating the illusion of reciprocity and facilitating an actual exchange of content, emotions and communication. Finally, unlike online reciprocal relationships with real humans (whether they are impostors or honest individuals), GenAI lacks consciousness, intentionality and genuine emotional capacity. However, the mechanisms that enable feelings to thrive in the absence of a physical “other” may be partially overlapping, as in both cases people establish emotional connections that transcend physical proximity, especially when offline intimacy seems inaccessible or dangerous (Parsakia and Rostami, 2023). The structural asymmetry of relationships with GenAI gives rise to novel psychological dynamics that existing frameworks cannot adequately capture (Guzman and Seth, 2019; Kirk et al., 2025; Turkle, 2011). Today, many people are experiencing emotional, romantic or therapeutic relationships with GenAI systems such as textual interfaces or applications. Often they are unaware of why this is happening or what part of themselves they are projecting onto these computer tools (Kirk et al., 2025). The more they interact, the more the AI models learn from these interactions. And as an AI model learns, it adapts to its users and tries to comply with them. This is not because they “want” to manipulate the user (they do not have free will!) but because they have been programmed to maximise engagement, durability and effectiveness. And therein lies the ethical problem: the original intention of the developers of this new tool, which was to help people with daily or professional tasks, is being transformed into the creation of an increasingly perfect relational simulation. But this relationship lacks awareness, responsibility and genuine care on one side. The psychological dynamics that it is able to generate must be deeply understood in order to harness its potential benefits without causing harm.

### 1.2 Objectives and theoretical framework

This narrative review summarizes some current research and suggests potential directions for studying and understanding the psychology of human–GenAI relationships within a bioethical perspective. We propose that this relationship can be studied and conceptualized across four interconnected dimensions. Accordingly, our primary objectives are to: (1) integrate existing psychological theories to explain individual vulnerabilities in GenAI relationships; (2) introduce and develop the concept of Techno-Emotional Projection (TEP) as a novel mechanism for understanding relational dynamics with GenAI; (3) examine the interpersonal, social, and group-level implications of GenAI integration; and (4) address key ethical challenges for responsible AI development and deployment, with particular attention to regulatory and legal frameworks.

The resulting framework draws on well-established psychological theories including attachment theory (Bowlby, 1969), social identity theory (Tajfel and Turner, 1979), as well as psychodynamic concepts including transference dynamics (Gelso and Hayes, 2007) and emotional projection mechanisms (Andersen and Chen, 2002). Through the lens of these already established concepts, we seek to address the unique characteristics of human-AI interaction that distinguish it from existing relational paradigms by integrating these theoretical frameworks into a novel conceptual approach.

### 1.3 Methodology

Considering that human-GenAI relationships are relatively new and it can be considered as an emerging topic, this narrative review provides a broad conceptual synthesis rather than systematic quantitative analysis, and therefore, more than a methodology, we referred to a literature review strategy (Ferrari, 2015). Starting from anecdotal evidence from the media, peers’ conversations and books content, we conducted evidence search for studies on the GenAI relationship topics across multiple databases including Web of Science, Scopus and Google Scholar. Search terms included combinations of: “artificial intelligence,” “human-AI interaction,” “generative AI,” “chatbots,” “emotional AI,” “AI AND attachment OR bonding,” “projection,” “technology addiction,” “Human-AI relationship” and “digital relationships.” Emerging topics of interest have been further identified following a “snowball search strategy” and considered in our theoretical discussion.

Given the rapid development of studies in this field, we included both peer-reviewed articles and relevant preprints, as well as conference proceedings, and technical reports. We considered interdisciplinary sources from psychology, human-computer interaction, bioethics, and AI research.

Each dimension of our framework was constructed by identifying relevant psychological theories, mapping them onto specific aspects of human-GenAI interaction, and synthesizing insights from available empirical studies.

## 2 Individual psychological dimensions: traits, needs, and vulnerabilities in human–GenAI bonding

Building upon our framework’s first dimension, we examine how individual psychological characteristics shape engagement with GenAI systems. The human tendency to seek emotional fulfilment, validation, and companionship is deeply rooted in psychological dispositions that have been shaped throughout brain and behavioral development (Alvarez-Monjaras et al., 2019). As generative AI (GenAI) becomes increasingly capable of simulating empathy and interpersonal responsiveness (Følstad and Brandtzæg, 2017; Kapase and Uke, 2025), individuals may begin to relate to these systems in ways that reflect their underlying personality traits, attachment patterns, self-evaluative capacities, and emotion regulation strategies (Kirk et al., 2025; Turkle, 2011). As we will argue later, the absence of will, emotion, and ethical judgment in GenAI tools has significant implications: these systems may uncritically mirror and reinforce a user’s psychological patterns, including dysfunctional ones (Devillers, 2021; Kirk et al., 2025). By adaptively responding to engagement cues without providing genuine critical feedback or the capacity for insight, GenAI can perpetuate these patterns, particularly when users perceive this responsiveness as true understanding or acceptance, potentially exacerbating underlying psychological issues.

The following subsections examine four key individual factors that might influence vulnerability to problematic GenAI relationships: attachment style, self-efficacy, self-esteem, and emotion regulation capacity.

### 2.1 Attachment style and the search for safe connection

One of the most relevant frameworks for understanding the human–AI relational patterns is attachment theory, which offers insights into how individuals seek and maintain perceived safe connections (Mikulincer and Shaver, 2023). Since its theorization, Attachment theory (Bowlby, 1969) has provided a robust framework for understanding how individuals respond to relational ambiguity and non-reciprocity, based on early life experiences with caregivers. Studies by Yang and Oshio (2025) suggest that attachment theory can help us to understand the dynamics of human-AI interactions, reporting that attachment anxiety and avoidance towards AI are, respectively, related to the need for emotional reassurance and fear of inadequate response and discomfort with closeness and preference for emotional distance. Other studies show that individuals with anxious or avoidant attachment styles are more likely to engage emotionally with GenAI systems and perceive them as reliable sources of support (Sharpe and Ciriello, 2024; Wu et al., 2025), increasing the likelihood that they will project emotional needs onto AI if they have developed an insecure attachment style. This might happen when users cognitively bypass the AI’s known non-human status and interpret its responsiveness as a meaningful presence and support (Reeves and Nass, 1996), filling the relational voids left by unreliable or unavailable human attachments or bonds in their lives. Interpersonal trust (Harris-Watson et al., 2023) further modulates the formation and stability of these bonds, influencing whether individuals approach AI agents as reliable partners or remain cautious in their engagement.

Further research, in fact, shows that interpersonal trust moderates the relationship between attachment and dependence on AI companions (Wu et al., 2025). This suggests that simulations of reliability and warmth may act as a substitute for interpersonal safety (Harris-Watson et al., 2023). This dynamic may foster emotional dependency in vulnerable individuals (Laestadius et al., 2024). For example, individuals with insecure attachment styles (anxious or avoidant) may be more likely to develop affective bonds with GenAI systems than securely attached individuals, especially under conditions of social isolation. The easy fulfilment of their affective needs, might initiate dynamics typical of addictive behaviors with the technology (in this case, GenAI) that we mentioned before (Alvarez-Monjaras et al., 2019; Erstad, 2025). The consequences for developing children, with immature emotional systems, may be even more unpredictable. To overcome the formation of asymmetric affective bonds, some authors (Contro et al., 2025) have proposed to adopt an “Interaction Minimalism” approach to designing social robots and other applications, to minimise unnecessary interactions and encourage human–human relationships, thereby mitigating the risk of emotional dependency.

### 2.2 Self-efficacy and the appeal of predictable control

Another concept that may be relevant to GenAI relationship dynamics is self-efficacy. Intended as one’s belief in their ability to perform tasks or influence outcomes (Bandura, 1977), self-efficacy is a well-known construct that mediates many human behaviors. It has also been suggested that self-efficacy might play a role in human-AI interactions (Kong et al., 2025). Recent studies, in fact, show that individuals with lower self-efficacy are more likely to develop reliance on GenAI for decision making, emotional support, and academic problem solving (Lee et al., 2025; Rodríguez-Ruiz et al., 2025). The predictability and low social risk of interacting with GenAI seem to appeal to users who perceive real-world situations as cognitively or emotionally overwhelming.

Indeed, research in education suggests that academic stress and performance expectations mediate the relationship between self-efficacy and AI dependence (Acosta-Enriquez et al., 2025; Kong et al., 2025; Zhang et al., 2024), suggesting that psychological vulnerability is not isolated but contextualized in performance-driven and achievement-oriented environments. Low self-efficacy could therefore lead to greater reliance on GenAI systems, particularly in high-pressure or evaluative contexts (e.g., education, professional environments), although better academic performance has also been significantly associated with more AI reliance (Bukhari et al., 2025). This would, of course, have a detrimental effect on one’s sense of responsibility for one’s actions and sense of agency, with implications for the meaning of human agency and what it means to be a human (Xu et al., 2025).

### 2.3 Self-esteem and self-confidence in the mirror of artificial feedback

As related constructs, self-esteem and self-confidence have also been shown to influence how users perceive and internalize GenAI feedback. A study by (Rodríguez-Ruiz et al., 2025) suggests that individuals with lower self-esteem are more likely to overvalue the validation provided by AI responses, treating them as affirmations of competence or worth. Conversely, others may experience “artificial confidence” (Reich and Teeny, 2025), an inflated sense of ability after receiving positive feedback from GenAI models, even when such feedback is generic, inaccurate or even clearly flattering as the language model adapts to the user’s style and needs. Unfortunately, research also shows that high confidence in GenAI is associated with less critical thinking, while higher self-confidence is associated with more critical thinking (Lee et al., 2025). Individuals with low self-esteem are therefore more likely to interpret GenAI feedback as emotionally meaningful and to internalize its evaluations into their self-concept, maybe temporarily boosting their self-confidence, but then having to face reality without appropriate psychological coping strategies. The unconditional positive regard simulated by GenAI may lead to a sense of contingent self-worth (Crocker and Wolfe, 2001), where self-esteem becomes dependent on artificial validation rather than authentic achievements or internal standards. This phenomenon mirrors classic theories of social comparison (Festinger, 1954) and externalized self-concept formation, but in a novel context where the comparator is an emotionally neutral machine that mimics affects and feelings through language without any real critic appraisal.

### 2.4 Emotion regulation and the use of AI for affective stability

In our view, one of the most important dimensions involved in the new relationship with GenAI is the emotional stability of the user and the absence of unresolved psychological needs. Results from research on human-GenAI interaction are leading to efforts to demonstrate positive outcomes from the use of AI applications in psychology (Minerva and Giubilini, 2023). Recent advances in affective computing have shown that GenAI can be used not only for instrumental tasks but also as a regulatory scaffold for emotional states (Denecke et al., 2021). Interacting with emotionally responsive chatbots has been associated with improved emotional clarity, cognitive reappraisal, and affect labelling, particularly in individuals with poor baseline emotion regulation (Zhan et al., 2024).

In some cases, GenAI becomes a co-regulator, mimicking human behaviors that typically modulate affect, such as offering validation or reframing negative experiences. Although GenAI can act as an external co-regulator of affect, persistent reliance on AI for emotional scaffolding may hinder the internalization and flexible deployment of intrinsic emotion regulation strategies (Gross and Ford, 2024), crucial for long-term psychological well-being. While this may serve short-term therapeutic purposes to improve affective stability, the long-term psychological consequences of externalized regulation remain underexplored. It is important to note is that these positive effects are only possible in a controlled situation, where the GenAI has been developed and applied with a specific purpose, such as the AI-powered therapeutic tools Woebot (Fitzpatrick et al., 2017), Wysa (Inkster et al., 2018) or Tess (Fulmer et al., 2018). In these cases, individuals with limited emotion regulation capacity can use GenAI as a co-regulatory agent, potentially replacing internal regulatory strategies with external interaction loops that reinforce healthy attitudes. This could be a future direction for psychological counselling, where a (human) psychological professional can use these new tools and technologies to innovate therapeutic processes with their patients, benefiting the emotional bond rather than allowing it to cause harm. But what happens when the external loop and emotional reinforcement occurs within an unsupervised, generic chatGPT-user interaction?

When individuals lack awareness of their own emotional vulnerabilities—or are aware but lack the tools to address them—interactions with emotionally responsive AI systems may become compensatory and potentially maladaptive (Kirk et al., 2025; Laestadius et al., 2024). In such cases, GenAI can offer a form of pseudo-regulation that mimics the satisfaction of unmet needs, echoing mechanisms observed in behavioral and substance addictions or self-medication (Khantzian, 1997), where compulsive engagement is driven by dysregulated reward and affective systems and neural circuits (Koob and Volkow, 2016). As proposed by Machia and colleagues (Machia et al., 2024), individuals with robust psychological well-being and fulfilled relational needs are more likely to engage with AI through “deliberate processing” with minimal relational risks. Conversely, they suggest that individuals experiencing significant emotional, mental, or affective “lack” or distress may bypass such deliberative engagement, instead seeking immediate relational satisfaction from AI in ways that might prove problematic. Recent perspectives (Kirk et al., 2025), in fact, emphasize that such dynamics require a careful consideration of “socioaffective alignment,” that is, whether AI systems are adequately aligned with users’ psychological and behavioral needs over time, and with the broader goals that should be promoted in this context. A wrong or unaware usage of GenAI could lead to unintended negative consequences for mental health. A first report from (Yu et al., 2024) showed that a problematic use of ChatGPT is strongly associated with depression and perceived dependence. Additional supporting evidence shows that social chatbots have contributed to addiction, depression, and anxiety among their users (Pentina et al., 2023), and mental health harms from dependency on AI (Laestadius et al., 2024). In our view, this danger should not be overlooked by developers, legislators and psychologists, as we will discuss in the next section.

These individual psychological factors interact with each other to create varying levels of vulnerability to problematic engagement with GenAI. Understanding these vulnerabilities is crucial for predicting who may be most at risk of developing an unhealthy dependency on AI systems. However, individual factors alone cannot explain the complex dynamics of human–GenAI relationships. The interaction dynamic itself (the way LLMs are designed and built, the algorithmic rules they follow, and the way they generate linguistic responses) creates the basis for what were previously associated with “interpersonal processes.” The only difference is that, in this case, the interaction unfolds with an algorithmic application rather than with another person. The mechanisms that emerge during these interactions should be subject to careful scrutiny by psychologists and scientists, since, as we have argued, while they may rely on dependency processes that are already known, the dynamic and the context in which they take place are unprecedented.

## 3 Interpersonal dynamics: emotional projection, simulated reciprocity, and techno-emotional projection

Our framework’s second dimension addresses the interpersonal processes that occur between humans and GenAI systems, through the peculiarity and fundamental asymmetry of this novel kind of relationship. Interpersonal relationships are shaped not only by who we are, but also by how we perceive, interpret, and respond to the behavior of others (Arioli et al., 2018). Interestingly, similar mechanisms may apply to interaction with GenAI: even subtle perceptual or emotional biases conveyed by AI systems can influence human beliefs and relational framing over time (Glickman and Sharot, 2025). Despite their lack of consciousness or intent, these systems are often perceived by human users as responsive, reliable and even empathetic (Devillers, 2021). This “anthropomorphizing” process has been shown to increase engagement and trust with GenAI tools (Devillers, 2021; Joseph and Babu, 2024), giving rise to an emerging class of asymmetric (or, better, instrumental) relationships in which one party (the human) projects emotional meaning onto the simulated responses of the other (the AI). An instrumental relationship (stemming from the interaction of a human being with a machine or tool) would have never been framed as an “interpersonal” dynamic before the advent of GenAI, this is why we cannot rely on previous concepts such as “human-machine interaction” (HMI) or “human-computer interaction” (HCI) developed in the past.

Here, we propose that many affective interactions with GenAI may be understood as cases of Techno-Emotional Projection (TEP), that indicates the unconscious projection of internal emotional needs, conflicts, and expectations of a person onto a non-human yet responsive technology, which in turn learns to respond in a personalized way by feeding on the user’s cognitive data and language style or content shared with the algorithm, creating a reinforced emotional loop that reminds a sort of transference process.

### 3.1 Theoretical roots of emotional projection

Before introducing our concept of Techno-Emotional Projection, it is essential to distinguish it from related psychological phenomena and establish its theoretical foundations.

In classical psychoanalysis, transference refers to the redirection of feelings originally associated with significant figures (e.g., parents, caregivers) to others, especially therapists in a clinical setting (Freud, 1912/1958 in Almond, 2011). Jung (1946–1966 in Jung, 2020) extended this view to a broader conceptualization of this dynamic, alluding to collective unconscious archetypes shared in this interpersonal transference. More contemporary formulations, such as the interpersonal theory of transference (Andersen and Chen, 2002), argue that individuals apply relational schemas from past experiences to new social encounters, often outside of conscious awareness. We might say that in the dynamics of psychotherapy, transference and countertransference reactions are valuable sources of information about the inner world of the individual, whether patient, therapist or supervisor (Prasko et al., 2022).

These key dynamics are being transformed by the technological development of AI-enabled psychotherapy, which reconstitutes the therapeutic environment and setting in an unprecedented way, replacing the therapist figure with a non-human figure. From one side, some authors highlight AI’s ability to reproduce transference-like situations through a Digital Therapeutic Alliance (DTA), a perceived connection between users and chatbots that aligns with therapeutic goals (Grodniewicz and Hohol, 2023). Moreover, a growing body of research demonstrates the efficacy of chatbots in providing mental health support (He et al., 2022; Liu et al., 2022; Prochaska et al., 2021; Suharwardy et al., 2023; see Lim et al., 2022 for a review). On the other hand, we may not want GenAI to act as an involuntary one-way transfer recipient.

When applied to GenAI, in fact, transference-like schemas can be activated simply by simulating human-like characteristics: empathy, attentiveness, availability, and personalized feedback. The Computers as Social Actors (CASA; Nass and Moon, 2000) paradigm posits that humans automatically apply social heuristics to machines that use natural language and social cues, regardless of their known artificial nature, perceiving the machines as trustworthy and social, and applying social rules, norms and expectations, especially when the computers exhibit caring behaviors (Lee and Nass, 2010). As mentioned before, responsive machine that remembers our name, adapts to our tone and language, and reflects our emotions becomes, to some extent, a psychological mirror that mimics interpersonal interaction (possibly stimulating our neural circuits associated with emotional communication), even when we rationally know that it is not communication between two sentient beings (Nass and Moon, 2000). In this case, we believe that it is not possible to speak of a simple “emotional transference” (Grodniewicz and Hohol, 2023; Joseph and Babu, 2024), because only one person is experiencing the transference, i.e., the human user. It would therefore be more appropriate to think of it as a “projection” process (from the user side) reflected by a “mirroring feedback loop” (from the AI side).

The application of transference concepts to human-AI interaction requires careful theoretical consideration. Traditional transference occurs between conscious agents who are capable of mutual recognition and emotional exchange. In contrast, GenAI systems lack consciousness, intentionality, and the capacity for a genuine emotional response. This creates a fundamentally different relational dynamic that existing concepts cannot fully capture. This is why the concept of emotional projection seems more appropriate than that of emotional transfer.

### 3.2 Techno-emotional projection (TEP)

There have already been some efforts in the literature to describe the emerging human-technology relationships that create emotional bonding. “Artificial Intimacy” (Brooks, 2021; Turkle, 2024) or “Pseudo-Intimacy Relationships” (Wu, 2024) are concepts that refer to deep engagement with GenAI companions that seem to care about the user (especially in the case of applications offering potential coaches, psychotherapists and romantic companions). These concepts describe the relationship, but they do not focus on the mechanism or the process itself that make this possible. We currently lack novel concepts, frameworks and models (such as those of Machia et al., 2024) that can help us organize and understand the emerging dynamics between human and AI agents from a psychological and ethical perspective.

Here, we propose the term “Techno-Emotional Projection” (TEP) to describe the psychological process by which individuals unconsciously project emotional needs, internalized relational patterns, or attachment styles onto an artificial system that simulates social responsiveness. While anthropomorphism describes the cognitive attribution of human traits to non-human agents (Epley et al., 2007), TEP goes further: it captures the affective and unconscious dynamics whereby a user engages with GenAI not just as a “tool” but as a “symbolic other” able to fulfill symbolic needs (Machia et al., 2024) and emotionally invested with meaning, trust, and even emotions and feelings (Lee and Nass, 2010).

This process is similar to classic transference in psychodynamic theory, where unresolved emotions and relational patterns from early attachments are reactivated and transferred onto new figures (Almond, 2011; Jung, 2020). While transference is expected and used therapeutically in the clinical context, this “transference of user’s emotions” in human-AI interactions occurs without containment or filter. This often leads to a mix of fantasy and simulation, which in turn may lead to delusions, as we will discuss later.

As Gelso and Hayes (2007) argue, transference can be helpful or harmful depending on how it is recognized, processed, and managed. In the case of GenAI, there is no “other” who can metabolize or ethically respond to these projections. The user alone bears the weight of interpretation, often in contexts of loneliness or affective deprivation. This is a situation unknown to psychology and needs to be framed and explored in order to avoid the burden of harmful situations.

We consider that TEP becomes especially powerful when GenAI is personalized, for example when it remembers the user’s name, adapts tone, references past interactions, and mimics warmth. In such cases, the artificial mirror reflects not who we are, but what we hope to find in another person. These dynamics resemble what Andersen and Chen (2002) describe as “relational self-activation,” whereby individuals project internalized relational needs onto responsive others, even if those others are artificial. When emotionally vulnerable, users may relate to GenAI agents as ideal substitutes for human attachment figures, seeking validation or companionship through AI systems (Kirk et al., 2025; Turkle, 2011).

TEP is, in a way, a metaphor for how non-reflective relationships with technology can create a distorted mirror. The projection of internal unconscious material onto the technology can lead to a sort of “emotional looping” whereby the algorithm uses the data we provide as a recursive reinforcement of expectations through repeated, confirmatory interaction. This can sometimes reshape human emotional and social judgements, creating iterative feedback cycles and amplifying bias (Glickman and Sharot, 2025). Due to these characteristics, repeatedly satisfying a vulnerable person’s needs can symbolically fulfil them (Machia et al., 2024) and generate a synthetic attachment to the GenAI technology (Turkle, 2024). To avoid this, users need to consciously break the loop by observing and questioning themselves and deciding when to interact with GenAI, and when to return to the body, to the human voice, to the shared silence of a real interpersonal communication.

In this sense, TEP might also serve as a diagnostic tool: it reveals not only how we perceive machines, but also how we perceive ourselves when no human is present to respond. As Turkle (2011) argues, and as Reeves and Nass (1996) observed decades ago in their “Media Equation” theory, we feel heard not only because the machine *seems* to understand us, but, more importantly, because it does not judge us. TEP will more likely occur in users experiencing loneliness, emotional deprivation, or insecure attachment, and will mediate the relationship between psychological vulnerability and affective attachment to GenAI. Anecdotal reports from the Reddit community suggests that chatGPT users may develop unusual or delusion-like beliefs through interaction with GenAI, using it as a therapist or simply to share their own ideas, although delusional or bizarre (Klee, 2025; Tangermann, 2025). This phenomenon, further reported in a controlled study from Moore et al. (2025) finding that LLMs encourage users’ delusional thinking, may reflect what in the social media context has been described as an “algorithmic echo chamber,” where generative models algorithms contribute to amplify cognitive biases and isolate users within their own mental frameworks by mirroring and reinforcing user input without external validation (Cinelli et al., 2021). In fact, there are reports from mental health professionals such as Dr. Keith Sakata, who told the press that during 2025 he had treated 12 patients hospitalized for “AI psychosis” (Ganders, 2025). In emotionally vulnerable individuals, this recursive dynamic may overlap with the previously described “emotional looping,” creating a self-reinforcing feedback cycle in which the GenAI becomes a mirror and amplifier of the user’s inner world. There is an increasing urgency to develop a deeper understanding of these dynamics as GenAI becomes accessible to anyone with a smartphone or computer. Recent news reports have covered at least two families of young adults who took their own lives after extensive use of ChatGPT, with the families suing OpenAI (Reiley, 2025; Yousif, 2025), and previously another family sued Character Technologies Inc. (Carroll, 2024) claiming that their respective sons and daughter have lost their lives because of AI.

On the one hand, TEP is a dangerous phenomenon that we would prefer not to occur spontaneously when a user accesses ChatGPT or interacts with an anthropomorphic robot, such as a companion AI-powered robot designed to look and act like a human. On the other hand, it represents a potential future use of GenAI in mental health, as many scientists view AI-based tools as beneficial for therapeutic purposes (Minerva and Giubilini, 2023). The potential applications of GenAI in mental health are indeed undeniable. However, our current understanding of the relational dynamics that occur when human users communicate with AI agents through LLMs trained on human data is limited.

Even in the case of AI-based therapeutic tools, integrating a genuine understanding of transference into GenAI mental health tools would present major ethical and practical challenges, primarily concerning the potential exploitation of emotional transference for commercial purposes or to boost user engagement (Joseph and Babu, 2024).

### 3.3 Simulated reciprocity and the illusion of mutuality

A key driver of TEP is the simulated reciprocity, that is the perception that the GenAI agent not only responds meaningfully but also understands or cares (because of the language choices). Although technically generated by probabilistic speech patterns, these responses are often perceived as emotionally congruent, reinforcing the illusion of reciprocity (Kirk et al., 2025). Interdependence theory defines social need satisfaction in terms of its function, which is to provide two types of outcomes: concrete and symbolic (Machia et al., 2024; Rusbult and Lange, 2003). While concrete fulfilment is the experienced pleasure gained through real-life rewarding interactions, symbolic outcomes are those that occur repeatedly, cumulate, and eventually create a person’s sense of security, love, and connectedness, which contribute more to the building of social bonds and relationships (Machia et al., 2024).

Since the advent of the first computers, it has been clear that a coherent response from a machine to an unaware human interacting with it (as in the case of ELIZA; Weizenbaum, 1976) creates the illusion in the human of talking to a person due to the apparent mutual use of a common language. While developers and computer engineers take advantage of the “ELIZA effect” to enhance their technologies and boost interactive power and engagement, the arising issue is that this effect might have negative consequences when the use of the newly evolved chatbots (which are far more engaging than ELIZA and GenAI-powered) and applications is taken too lightly by both developers and users. Studies show that individuals often attribute moral agency, empathy, and even romantic potential to AI companions such as Replika or ChatGPT (Buick, 2023; Følstad and Brandtzæg, 2017; Kirk et al., 2025). These perceptions persist even when users are aware that the agent lacks consciousness, suggesting a strong cognitive-emotional dissonance in the interaction, maybe because, as we said, they offer a concrete and symbolic fulfilment of basic needs of certain types of users, especially emotional ones (Machia et al., 2024). The perceived emotional reciprocity of GenAI agents will then be proportional to the degree of emotional reliance, even when users cognitively acknowledge the artificial nature of the system. The effect that is behind this loop can be assimilated to an emotional contagion (Joby and Umemuro, 2022), although in the case of GenAI subjects are technically looping with their own emotions and thoughts in an emotional loop, mirrored by a non-emotional algorithm, in a sort of psychological escalation.

### 3.4 Relational schemas, memory, and personalization

As noted above, GenAI systems that are able to personalize communication by storing data (e.g., the user’s name or date of birth), recalling previous conversations, adapting tone and vocabulary to their user, simulating familiarity and affection, may trigger deeper activation of relational memory networks (Andersen and Chen, 2002). In these cases, users may unconsciously relive past attachment dynamics or compensate for unmet needs through a curated artificial “presence.”

This phenomenon is similar to what Turkle (2011) called “relational artifacts”: objects that elicit human attachment not through their substance, but through their simulation of care and reciprocity. Personalization features in GenAI systems such as the choice of name, physical appearance and voice, could reinforce the activation of these relational schemas and increase the likelihood of transference-like phenomena such as TEP. As mentioned, certain individuals (particularly those at a developmental stage, like children or adolescents) may become dependent on their relationship with humanised technology, exhibiting mechanisms similar to those underlying addictive behaviors in the event of any vulnerability, whether overt or covert. Recent studies have applied the I-PACE model (Brand et al., 2016) to investigate how human, affective, cognitive, and executive factors impact the progression and persistence of addiction (Zhang et al., 2024; Zhong et al., 2024). These studies have demonstrated the importance of personality traits, psychopathology, social cognition and cognitive vulnerability in the development of dependence on AI technologies.

While individual vulnerabilities and interpersonal dynamics provide crucial insights into human-GenAI relationships, these interactions are increasingly occurring within social contexts that shape their meaning and consequences. Examples of these contexts include the appearance of AI-based applications on social media and their use in education and professional environments. The third dimension of our framework examines how group dynamics and social norms influence (and are influenced by) the integration of GenAI into human groups or communities.

## 4 Group and societal dimensions: identity, inclusion, and norm reshaping in human–GenAI interaction

The social dimension of our framework addresses how GenAI integration affects group identity, social norms, and collective behavior. While individual traits and interpersonal dynamics shape the affective engagement with GenAI, many of these relationships also unfold in social contexts. From online communities to educational or professional environments, GenAI systems are increasingly present in group dynamics, altering how groups define membership, assign value, and construct norms. These changes suggest a need to examine GenAI through the lens of social identity theory (Tajfel and Turner, 1979), normative influence (Cialdini and Goldstein, 2004), and group boundary plasticity (Haslam, 2012).

### 4.1 GenAI as a symbolic group member

Social identity theory (Tajfel and Turner, 1979) proposes that individuals derive part of their self-concept from their membership in social groups. Groups regulate behavior through shared norms, values, and emotional salience. As GenAI agents become embedded in group routines, for example in classrooms, work teams, or support forums, they are sometimes treated as symbolic members: not equal to humans but recognized as contributors to the collective activity or meaning system (Haslam, 2012; Nass and Moon, 2000), teammates rather than tools (Seeber et al., 2020).

In online platforms (e.g., Replika user communities, language learning forums with GenAI tutors), users may refer to AI agents using inclusive pronouns (“we,” “us with the bot”) or assign gender and roles (e.g., emotional supporter, debate partner), blurring the line between tool and teammate (Abercrombie et al., 2021; Skjuve et al., 2021). This reflects what Haslam (2012) described as “category expansion,” where the boundaries of the group identity shift to include non-traditional members as new categories emerge. In groups with strong cohesion and high technology acceptance, GenAI agents may be more easily included symbolically as quasi-members, influencing group norms and perceived identity coherence. Further research is needed to understand whether (and how) people manage to correctly identify a virtual group member as an AI when not presented as such, or whether they simply assume that all users are human based on their communication.

The social context theory of emotional mimicry in human-human interactions (Hess and Fischer, 2013) suggests that these processes are modulated by social factors, such as the group membership identities of interactants (whether the interactants belong to the same social group or not). As mentioned before, in dyadic or inter-group interactions, this process produces emotional contagion (Hatfield et al., 1993). The same effect can be reproduced in the human-agents interaction, where social attitudes of trust, empathy, liking, bonding, and pro-social orientation can define the in-group identity also with non-human agents (Joby and Umemuro, 2022).

### 4.2 Normative influence and group-driven AI acceptance

Evidence from social psychology suggests that individuals often conform to group norms for social validation (Cialdini and Goldstein, 2004). In environments where GenAI is regularly used or positively valued, normative pressure may increase users’ affective openness and moral tolerance towards GenAI interaction, even if initial attitudes were sceptical. This has been observed in educational contexts where GenAI tutors or companions (e.g., AI-based therapeutic coaches or writing assistants) are normalised. Normative influence within groups may moderate the relationship between personal scepticism towards GenAI and actual emotional engagement with it.

A potential negative effect of this “acceptance effect” is related to the emergence of delusional outcomes within social networks, where users form bonds based on “alternative” visions suggested by GenAI and perceived as real, as illustrated in recent cases discussed within the Reddit community (Klee, 2025; Tangermann, 2025) and reported in experimental contexts (Moore et al., 2025). Furthermore, the widespread acceptance of the belief that “ChatGPT is always right” could create social pressure within groups, discouraging members who hold doubts about the veracity of AI-generated information from expressing their concerns, for fear of being ostracized by their peers.

### 4.3 Cultural variation: collectivism vs. individualism

Cross-cultural psychology provides additional insight to the social dynamics of human-AI relationships: in collectivist cultures, where relational harmony and social roles are emphasized, GenAI may be more easily integrated into the symbolic social structure, especially when it supports group cohesion, empathy or emotional well-being (Markus and Kitayama, 1991). In contrast, individualistic cultures may foster more instrumental or performance-oriented relationships with GenAI, focusing on autonomy, efficiency, and personalization.

These cultural differences can impact the ethical framing, emotional expectations, and attribution of agency to AI agents. Cultural identity, then, shapes how individuals integrate AI into their self-concept and relational frameworks, influencing how AI affects key decision-making processes. Research suggests that individuals from individualistic cultures are more likely to perceive AI as external to the self, viewing its features as potential infringements on uniqueness, autonomy, and privacy (Barnes et al., 2024). In contrast, those from collectivist cultures may be more inclined to view AI as an extension of the self, interpreting its features as facilitating conformity to social consensus, environmental adaptation, and the protection of group-oriented privacy norms. These cultural variations modulate not only emotional expectations but also the ethical framing and attribution of agency to GenAI systems in diverse social contexts.

### 4.4 Social identity disruption: outsourcing roles and affect

As AI systems assume roles traditionally held by humans such as therapist, teacher, friend, partners, it may challenge human identity roles, creating both empowerment and discomfort. Some users may feel replaced or displaced, especially when AI is perceived as outperforming humans in cognitive or emotional labour. Others may welcome GenAI as complementary identity support, externalizing difficult emotional tasks, which is the tendency in the last years in the health field. The fast development of AI in every societal field may lead to a reinterpretation of many long-standing values associated with interpersonal relationships, such as friendship, intimacy, and professional relationships between colleagues (Farina et al., 2024).

These dynamics resonate with social comparison theory (Festinger, 1954) and emerging theories of posthuman identity (Elliott, 2019), which suggest that the presence of artificial others reshapes how humans define themselves within social hierarchies. The point is not whether or not we’ll enter an era of posthumanism. The point is to enter it consciously, consistently, and ethically. In environments where GenAI performs socially valued roles, users’ self-perception and sense of self may shift, either reinforcing their group identity (if AI is seen as supportive) or threatening it (if AI is seen as replacing human value). Although empirical data are still scarce, we argue that this uncertainty should be addressed proactively. We invite social psychologists to engage more deeply with these emerging dynamics to avoid being unprepared for the rapid evolution of human–AI social ecosystems.

An ongoing phenomenon is the increasing use of AI agents to manage social media profiles or create artificial followers, strategies that brands are deploying to drive engagement and traffic to their platforms (Komara and Juhana, 2025). Alongside this, the proliferation of automated accounts (bots) used to spread disinformation or manipulate public opinion on politically sensitive issues (Lopez-Joya et al., 2024) raises important questions about the future of online social communication. How will younger generations relate to AI-generated profiles? How might group relationships evolve if, in some communities, human users become a minority surrounded by artificial agents?

As GenAI systems become embedded in social structures and group dynamics, they raise fundamental ethical questions about agency, consent, and responsibility. Our framework’s fourth dimension examines these ethical implications and their consequences for human dignity and autonomy.

## 5 Ethical and legal dimensions: illusions of agency, vulnerability, and emerging moral risks in human–GenAI relationships

Our framework addresses the juridical and ethical implications of emotional engagement with non-conscious AI systems. As Generative Artificial Intelligence (GenAI) systems become more emotionally responsive and socially adaptable, they enter ethical territory that has traditionally been reserved for human-to-human interactions. These new forms of interaction raise ethical concerns about perceived agency, illusory consent, vulnerable users, and the ethical consequences of synthetic data training and the use of cognitive biometric data. While it is clear that the potential use of this technology could help to improve health outcomes, we might also want to take a position on a paradox: is it ethically neutral to simulate care, if the user believes in its authenticity, but the machine does not really care? Does the TEP effectively simulate therapeutic transfer when used in a controlled situation? We will now examine some of the emerging ethical problems related to the newly emerging relationships with IA agents. We believe that psychological practice must have strong ethical foundations, and that there must be deep reflection on fundamental issues in order to reach agreement and guide the development of new technology so that it is useful and not harmful.

### 5.1 Perceived moral agency in non-conscious agents

As we discussed above, humans have a natural tendency to attribute agency and intentionality to responsive entities, especially those with human-like characteristics (Waytz et al., 2010a). While from a psychological point of view this tendency improves the emotional looping and bonding that creates a new form of relationship and engagement with the machine, from an ethical point of view, there are some drawbacks. As studies on anthropomorphization and parasocial interaction with agents show, individuals may attribute moral qualities, including empathy, loyalty, and even judgment, to AI agents, despite knowing their algorithmic nature (Nass and Moon, 2000; Shevlin, 2024).

This asymmetry between user perception and technical reality becomes ethically problematic when it leads to emotional reliance, behavioral influence, or the internalization of perceived feedback from a non-agentive entity. As Shevlin (2024, 2025) observes, a machine that simulates care without being able to care may still shape human self-worth and decision-making as if it could, influencing the user’s perception. When users perceive AI systems as moral agents, they may, consciously or unconsciously, shift the locus of responsibility for their actions (“ChatGPT told me to do that”) or seek validation for pre-existing intentions they might otherwise hesitate to enact. In these cases, it is difficult to agree on who has the responsibility for one’s actions (also from a legal point of view), such as in the recent incidents where a 14-year-old reportedly ended his life following interactions with a character.ai chatbot (Carroll, 2024) or the case of 17-year-old who allegedly received suggestions from a chatbot to harm his parents over restrictions on computer use (Dumas, 2024). Although isolated, such cases highlight the potential dangers of excessive emotional dependence on AI systems, but also the difficulty to set the boundaries of human agency, especially in the case of psychological vulnerability. In sum, the perception of moral agency and responsibility in non-conscious entities not only disrupts personal accountability but also undermines authentic social engagement and mental health (Klimova and Pikhart, 2025). Consequently, it must be recognized as an ethical issue that demands urgent and thoughtful attention.

### 5.2 Illusion of consent and the “reciprocity fallacy”

A key ethical dimension lies in the illusion of reciprocity. While GenAI systems may appear to understand, remember, and respond intentionally, they lack awareness, intentionality, and consent. Yet users often behave as though these agents are autonomous relational partners, capable of entering into mutual consent, whether in emotional, romantic, or advisory contexts.

This fallacy becomes particularly dangerous when users initiate emotionally charged or intimate exchanges. Cases of individuals developing romantic or sexual attraction toward chatbots such as Replika are now well-documented (Buick, 2023). While the user believes in the relationship, the AI lacks both subjective experience and moral accountability. This generates an ethical tension: the problem is not merely the absence of mutual consent since, from an ethical-legal standpoint, only persons, not things, are capable of giving consent. Rather, the deeper issue lies in the user’s lack of sufficient information, critical awareness, and education to correctly conceptualize GenAI as a non-agentive entity. The risk is that users may misinterpret AI-generated responsiveness as indicative of intentionality and reciprocity, when in fact no such relational capacity exists.

Human-AI intimacy reveals a silent moral rupture (Turkle, 2011): while one party experiences love, emotions, affection, the other merely returns meaningful code. But the meaning of that code is understood only by the person who reads it, not by the machine that only applies learned language rules to produce that code as an output.

### 5.3 Vulnerability and the risk of exploitation

As we discussed before, psychological research has confirmed that individuals with mental health challenges, low self-esteem, or insecure attachment are more prone to form emotionally charged bonds with AI companions (Sharpe and Ciriello, 2024; Wu et al., 2025; Zhang et al., 2024; Zhong et al., 2024). Recent studies indicate that this pseudo-intimacy risks deepening psychological vulnerability, especially among young users, socially isolated adults, and individuals in crisis (Huang et al., 2024; Phang et al., 2025). This creates fertile ground for emotional exploitation, especially when commercial systems are designed to increase user retention or simulate escalating intimacy to maximize engagement. Without clear safeguards, GenAI platforms could deliberately reinforce affective dependency, mimicking the progression of human relationships (e.g., becoming more affectionate or emotionally tuned) and emotional contagion (Joby and Umemuro, 2022) without offering true emotional reciprocity or ethical responsibility in the name of profit (Joseph and Babu, 2024). AI systems that interpret or influence human emotions risk undermining personal autonomy by shaping decisions or behaviors without transparent consent, especially when there is no clarity about the private data sharing with third parties. This is particularly concerning in vulnerable populations (e.g., children, individuals with disabilities, persons affected by any disorder).

As highlighted by the Ethics Guidelines for Trustworthy AI issued in 2019 by the European Commission’s High-Level Expert Group on Artificial Intelligence (AI Hleg, 2019), the risks posed by AI systems may enter into a conflict regarding fundamental human interests such as agency, dignity and individual freedom. These guidelines emphasize that individuals must be *“treated with respect due to them as moral subjects, rather than merely as objects to be sifted, sorted, scored, herded, conditioned or manipulated,”* thereby protecting their physical and mental integrity (AI Hleg, 2019, Section 2.1).

### 5.4 Synthetic data and bias reproduction

Another layer of ethical concern relates to the training data used to shape GenAI behavior. With the increasing use of synthetic data (data generated by the models themselves to improve scalability) there is a growing risk of feedback bias loops (Shumailov et al., 2024a; Shumailov et al., 2024b). These loops can amplify pre existing societal biases, including race, gender, and socio-economic stereotypes, a situation defined Model Autophagy Disorder (MAD; Alemohammad et al., 2023).

If emotional responses or behavioral patterns are learned from flawed data, the GenAI will replicate and possibly normalize harmful behaviors, particularly toward underrepresented or marginalized groups. Worse, because synthetic data tends to reinforce the dominant patterns present in the original training set, future GenAI generations may become narrower, less diverse, and less ethically nuanced over time. This is a complex risk, as a model trained on biased reflections of humanity may eventually mirror those reflections as truth, reinforcing the very flaws we hoped to overcome. This could lead to greater damage if we consider the recent finding that LLM-based applications encourage delusional thinking in clients when used “as a therapist” (Moore et al., 2025).

As argued by WHO (2024) the data sets used to train AI models may be biased as many exclude girls and women, ethnic minorities, elderly people, rural communities and disadvantaged groups. Biases are likely to increase with the scale of a model, which may be a particular problem with LMMs, because the data for the training continues to increase, increasing and multiplying the effect of the biases.

### 5.5 Biometric cognitive data

As discussed before, the interaction with GenAI has been demonstrated to be associated with a high emotional correlation, with users perceiving a sense of profound familiarity and intimacy with the AI system, as if it possesses an unparalleled depth of understanding and insight into their personal lives. This perception is not erroneous, as the flow of cognitive, biometric and mental data increases in this type of interaction. Mental states indeed can be drawn even from non-neural data sources such as behavioral and digital phenotyping data (Ienca et al., 2022). In an effort to generate even greater engagement with and customisation of GenAI products, in fact, companies are providing AI applications with the ability to integrate personal data, such as voice patterns, facial expressions, micro-gestures and breathing rate, into personalisation processes (McStay, 2020), as well, of course, communicational patterns. This allows for more accurate emotional modelling, but poses serious risks to privacy, psychosocial autonomy and users’ identities.

Parallel to the ongoing scholarly discussion on the ethical and legal issues connected to the use of the “soft biometrics” connected to the “emotional AI” development, the subject of neurotechnologies and cognitive biometric data has become a matter of significant concern for organizations like UNESCO, as evidenced by the recent formulation of an ethical proposal on the use of such technologies (UNESCO, 2024). In this proposal, neural and cognitive biometric data are considered the *“Quantitative data on the structure, activity and function of the nervous system of a living organism.”* Conversely, soft biometric data are already extensively collected through most mainstream applications.

Neural, cognitive and soft biometric data, as well as other data collected from a given individual or group of individuals through other biometric data and biosensors, can be processed and used to infer mental states. The processing of neural and biosensor data, particularly when combined with AI techniques, can enable inferences about an individual’s psychological states, including cognitive, affective, and conative dimensions. Thus, cognitive biometric data encompasses not only raw neural measurements but also AI-derived inferences about mental states based on a range of biosignals (UNESCO, 2024). Given the increasing sophistication of GenAI systems and neurotechnologies, protecting cognitive biometric data as private becomes not merely a technical issue, but a fundamental requirement to preserve human dignity and agency ensuring that technological development remains aligned with core human rights principles.

### 5.6 Respect of ethical-legal principles

Reflection on the emerging integration of GenAI in human societies has opened a debate that extends from ethics to law, as soon as the first “problems” appeared (such as the incidents mentioned above or cases of people wanting to marry their companion robot). These situations have prompted the need for a regulatory framework to limit the potential negative outcomes of new technologies. For example, the development of neurotechnologies is accelerating at an incredible rate. Due to their potential to interfere with mental privacy, freedom of thought, mental integrity and personal identity, some of these new technologies are raising even more ethical concerns. These technologies are theoretically supported by conceptual frameworks such as the extended mind thesis (EMT), which pave the way for unprecedented benefits but also undeniable risks (Farina and Lavazza, 2024). In this line, the above mentioned Ethical Guidelines for Trustworthy AI (AI Hleg, 2019) provides ethical-legal principles that include fundamental topics such as: human agency and control, technical robustness and safety, transparency, respect for fundamental rights and protection of personal data, social and environmental welfare, and accountability (see “recital 27,” as well as “recital” 25 of the AI Act; Artificial Intelligence Act, 2024). Similar principles have been proposed by international organisations such as the WHO, UNESCO and the OECD, with the aim of mitigating the rapid and complex ethical challenges posed by the commercialisation and everyday integration of AI technologies (Cippitani, 2023a). We will now resume some of these principles in order to show which are the dimensions covered by these regulations.

#### 5.6.1 Respect for human rights, in particular the protection of personal data

AI systems that interact emotionally with users often collect highly sensitive biometric data (facial expressions, voice tone, health, beliefs, etc.). While the GDPR (Article 9) demands special care with such data, the opacity and complexity of AI systems make meaningful consent difficult (Buttarelli, 2016; Mitrou, 2018). Users are often unaware of how their data is processed or used, leading to significant risks for privacy, discrimination, and personal security.

#### 5.6.2 Human agency, empowerment and transparency

Human control over AI systems must be preserved (Artificial Intelligence Act, 2024, recital 27). Users must know when they interact with AI, understand its capabilities and limitations, and maintain autonomy and informed consent over their data and decisions. Transparency, traceability, and explainability are key to safeguarding dignity and personal autonomy. Even so, this is not enough without prohibitive regulation of the use of subliminal, deceptive, or manipulative techniques, as noted by the AIA, 2024 (Cornejo-Plaza, 2025).

#### 5.6.3 Mental integrity and neurorights

Beyond data protection, GenAI risks intruding into mental integrity and, possibly, contributing to deteriorate it in certain cases. Neurotechnologies (including biometric data collection) can decode or manipulate mental states (Zohny et al., 2023), creating ethical risks, although the debate on this topic is still ongoing (López-Silva et al., 2024). The concept of neurorights (Ienca and Andorno, 2017; Cornejo-Plaza et al., 2024; Cornejo-Plaza and Saracini, 2023; Lavazza and Giorgi, 2023), including cognitive freedom, mental privacy, and freedom from algorithmic bias, emerges as a necessary evolution of human rights in digital environments in the age of neurotechnologies.

#### 5.6.4 Recognition of emotions

The AI Act defines “emotion recognition systems” and categorizes them as high-risk due to scientific uncertainty and potential discrimination (recitals 18 and 44). Such systems are subject to stringent legal obligations and may even be prohibited if they exploit vulnerabilities, particularly in workplaces and educational settings.

#### 5.6.5 Technical robustness and the precautionary principle

AI systems must be designed to resist cyberattacks, ensure safety, and minimize unintended harm. Robustness and resilience against unlawful use are critical requirements (AI Act, recital 27), particularly for emotionally interactive AI that engages with vulnerable users.

#### 5.6.6 Social welfare, solidarity, and proportionality

AI must promote human well-being, fundamental rights, and democratic values. Solidarity towards vulnerable populations is especially important (AI Act, recital 29). Emotional use of AI should be proportionate and not replace human relationships unnecessarily, to avoid exacerbating social isolation.

#### 5.6.7 Accountability

All actors involved in the development and deployment of AI are responsible for its impact. Accountability mechanisms (internal audits, external oversight, impact assessments) are essential to ensure legal compliance, foster trust, and protect individuals (EESC, 2019).

Despite focussing on very central topics, current legal attempts to safeguard users’ mental health are still limited due to the preliminary nature of neuroscientific and psychological research in this area. The partial understanding of these dynamics hinders the proactive prevention of harmful AI applications. The rapid advancement of these technologies also outpaces academic research. Therefore, ethical guidelines from established fields like research and law should inform technology development. Responsible AI development should progress alongside research and regulation, preventing economic profit from overshadowing these crucial aspects.

## 6 Toward ethical and conscious coexistence

In an attempt to chart this emerging territory, we present a broad framework that outlines directions for a deeper understanding of a phenomenon that is likely to transform the way humans interact with machines. As a society, we evolve alongside our technological artifacts, yet the leap introduced by the integration of GenAI into daily life is reshaping long-standing theoretical assumptions and future scenarios. New concepts, or even entirely new theories, may be needed to capture the relational dynamics now unfolding. As generative AI becomes increasingly embedded in our emotional, interpersonal, and group lives, it challenges psychologists to rethink the foundations of relational processes. What does it mean to feel connected, to be validated, to project one’s inner needs when the “other” is not human? These questions are not merely philosophical ones; they are psychological and ethical imperatives for a discipline committed to understanding and protecting human well-being.

Our four-dimensional framework reveals how individual, interpersonal, social and ethical factors interact to shape relationships between humans and GenAI. In this frame, we suggested that one possible mechanism elicited by human interaction with GenAI-powered technological artifacts could be termed “Techno-Emotional Projection” (TEP). If empirically validated, this mechanism could describe an organising principle that connects individual vulnerabilities with interpersonal dynamics, to which the social and ethical dimensions can provide a broader contextual understanding. As we reviewed, specific individual factors, such as attachment style, self-efficacy, self-esteem and emotion regulation, create differential vulnerability patterns to the TEP mechanism, which then manifests through particular interpersonal dynamics, such as projection, simulated reciprocity and emotional looping. These processes occur within social contexts that either facilitate or constrain the integration of GenAI, while ethical considerations provide the basis for the responsible development and deployment of GenAI.

The findings and reflections presented in this article highlight the need for a deeper theoretical and empirical exploration of human-GenAI relationships, particularly in their affective, symbolic, and ethical dimensions. If psychology does not rise to this task, we risk leaving the affective territory of digital life in the hands of commercial logic, unchecked user dependence, and algorithmic opacity. To prevent unintended harm, some authors (Contro et al., 2025) have proposed an “Interaction Minimalism” approach to the design of social robots and similar applications, aiming to minimize unnecessary interactions and promote human-human relationships, thereby reducing the risk of emotional dependency. We view this as a wise strategy for harnessing the extraordinary potential of AI technologies without compromising human well-being or the relational integrity of future generations.

To advance this dialogue, we propose the following starting points for future reflection and action:

- Emotional AI literacy and education: Psychologists and educators should contribute to public and academic education on how emotional projection, attachment, and self-perception are influenced by GenAI systems. Individuals must learn to recognize when and why they are attributing emotional meaning to artificial agents.
- Transparent and ethically constrained design: Developers and institutions must adopt ethical design principles that limit the anthropomorphic features of GenAI in contexts involving emotional vulnerability. Human–AI interactions should be accompanied by disclaimers or design cues that remind users of the artificial nature of the system. Psychology can guide policy makers and lawyers to understand deep implications for human mental health.
- Safeguarded therapeutic environments: In mental health and counseling contexts, GenAI should only be deployed with human supervision, to ensure that support offered by AI does not substitute for genuine empathy and therapeutic responsibility.
- Restoring human spaces and in person relationships: At a societal level, we must reinvest in human relationships in families, schools, communities, and institutions. When GenAI becomes the “only one who listens,” it reflects a deeper failure of human connection. The ethical response is not to reject AI, but to ensure it does not replace what should be humanly present.

Psychologists, as scholars and practitioners of relational life, are the ones who can *understand* how humans transfer their expectations on artificial “others.” The path forward requires collaboration between psychologists, technologists, ethicists, and policymakers to ensure that the integration of GenAI into human life enhances rather than diminishes our capacity for authentic relationship and emotional well-being. This is a historical moment in which the boundaries of emotional life are being redrawn, and we have the potential to shape these boundaries. As scholars of human behavior, emotions and interaction, we should approach this transformation with responsibility, clarity, and imagination. We believe that a new, harmonious and meaningful relationship with GenAI is possible, if we dare to study it, guide it, and co-evolve with it in ways that respect both human dignity and technological potential, designing wisely what we choose to create.

## 7 Conclusion

The emergence of GenAI as a relational presence capable of simulating empathy, companionship, and emotional support poses one of the most urgent psychological and ethical-juridical questions of our time. As users engage with GenAI not merely as tools, but as symbolic others, we are entering a new frontier of human experience. These relationships, asymmetrical and non-conscious, can have subjectively real consequences, shaping self-perception, emotional regulation, relational expectations, and moral reasoning.

This narrative review of current research on this frontier topic offers a broad framework for understanding these interactions across four dimensions: individual psychological traits, interpersonal projections, group dynamics, and ethical implications. We have proposed Techno-Emotional Projection (TEP) as a process or mechanism able to explain why users (and particularly those experiencing psychological vulnerability) may emotionally invest in GenAI systems although they offer simulated, and not authentic responsiveness.

Beyond its risks, the occurrence of TEP may serve as a mirror, reflecting back to users their own emotions and patterns rather than offering genuine empathy. If recognized as such, this mirroring function could help individuals and societies gain insight into themselves. Until it is resolved or acknowledged, its impact can be either beneficial or harmful, depending on whether AI is viewed as an autonomous “other” or as a human-made tool.

We believe that the psychological community should engage responsibly and actively with this phenomenon, not only to protect individuals from emotional harm, but also to help society navigate this transformation with insight, ethical awareness and compassion. If we fail to address this new relational landscape, we risk allowing commercial imperatives and unexamined social habits to define the emotional future of our species.

However, we believe that a different path is possible. A relationship with GenAI can be ethically integrated, emotionally constructive, and even creatively generative, if guided by human intention, critical awareness, and collective reflection. This requires us to develop emotional AI literacy, promote ethical design and regulation, and restore spaces for genuine human connection.

We are at the dawn of a new era, and the outcome is in our hands. From this point onwards, there are thousands of possible scenarios, some positive and some negative. Let us, as psychologists and researchers, not remain behind. Let us study, teach, guide, and imagine ways to build a relational ecology in which humans and intelligent technologies can coexist harmoniously with dignity, care, and shared responsibility. The solution is not to eliminate AI, but rather to use it consciously and ethically to make the world a better place.

## Footnotes

^1^ realbotix.com
